# Analyses in zebrafish embryos reveal that nanotoxicity profiles are dependent on surface-functionalization controlled penetrance of biological membranes

**DOI:** 10.1038/s41598-017-09312-z

**Published:** 2017-08-21

**Authors:** Ilkka Paatero, Eudald Casals, Rasmus Niemi, Ezgi Özliseli, Jessica M. Rosenholm, Cecilia Sahlgren

**Affiliations:** 1 Department of Cell Biology, Biozentrum, University of Basel, Basel, Switzerland; 2 Faculty of Science and Engineering, Cell Biology, Åbo Akademi University, FI-20520 Turku, Finland; 3 Turku Centre for Biotechnology, Åbo Akademi University and University of Turku, FI-20520 Turku, Finland; 4 Pharmaceutical Sciences Laboratory, Faculty of Science and Engineering, Åbo Akademi University, FI-20520 Turku, Finland; 5 Department of Biomedical Engineering, Technical University of Eindhoven, 5613 DR Eindhoven, The Netherlands; 6 Institute for Complex Molecular Systems, Eindhoven University of Technology, Eindhoven, The Netherlands

## Abstract

Mesoporous silica nanoparticles (MSNs) are extensively explored as drug delivery systems, but in depth understanding of design-toxicity relationships is still scarce. We used zebrafish (Danio rerio) embryos to study toxicity profiles of differently surface functionalized MSNs. Embryos with the chorion membrane intact, or dechoroniated embryos, were incubated or microinjected with amino (NH_2_-MSNs), polyethyleneimine (PEI-MSNs), succinic acid (SUCC-MSNs) or polyethyleneglycol (PEG-MSNs) functionalized MSNs. Toxicity was assessed by viability and cardiovascular function. NH_2_-MSNs, SUCC-MSNs and PEG-MSNs were well tolerated, 50 µg/ml PEI-MSNs induced 100% lethality 48 hours post fertilization (hpf). Dechoroniated embryos were more sensitive and 10 µg/ml PEI-MSNs reduced viability to 5% at 96hpf. Sensitivity to PEG- and SUCC-, but not NH_2_-MSNs, was also enhanced. Typically cardiovascular toxicity was evident prior to lethality. Confocal microscopy revealed that PEI-MSNs penetrated into the embryos whereas PEG-, NH2- and SUCC-MSNs remained aggregated on the skin surface. Direct exposure of inner organs by microinjecting NH_2_-MSNs and PEI-MSNs demonstrated that the particles displayed similar toxicity indicating that functionalization affects the toxicity profile by influencing penetrance through biological barriers. The data emphasize the need for careful analyses of toxicity mechanisms in relevant models and constitute an important knowledge step towards the development of safer and sustainable nanotherapies

## Introduction

Mesoporous silica nanoparticles (MSNs) are widely used for drug delivery applications, due to their unique properties, including nano-sized pores for loading of both small molecules and large biomolecules (proteins, nucleic acids), good biocompatibility and biodegradability^1^. MSNs are flexible in terms of surface functionalization strategies and offer vast opportunities for chemical modifications to influence biodistribution, drug release, efficacy and biodegradation^1^. There is therefore an urgent need to understand how different surface functionalizations affect biocompatibility and safety of MSNs or any other nanoparticulate system. Understanding the mechanisms of interaction between nanoparticles and biological systems at the molecular scale will provide important information to move toward safer nanotherapies by providing guidelines for rational design of materials.

The interaction between biological entities and nanoparticles is mediated by their respective surfaces. In this context, surface charge is recognized as a key parameter determining NPs interaction with the biological membranes, thus influencing cellular internalization, opsonisation, immune responses as well as and toxicity^2, 3^. It is known that positively charged macromolecules display an enhanced toxicity profile as compared to their neutral and negative counterparts^4, 5^. Hoshino *et al*.^6^ showed that more positively charged ZnS-coated CdSe nanocrystals Quantum Dots (QD) displayed higher cytotoxity using nanocrystals surface modified with carboxylic acids (Mercaptoundecanoic Acid, MUA), polyalcohols (thioglycerol), and amines (cysteamine) with zeta-potentials ranging from −58,75 mV in the case of MUA-QD to +40,52 mV for cysteamine-QD. Goodman *et al*.^7^ demonstrated that quaternary ammonium (positive zeta-potential) functionalized 2 nm core AuNPs were moderately toxic, whereas carboxylate-substituted NPs (negative zeta-potential) were quite nontoxic. For silica nanoparticles, non-functionalized NPs have in general been found to be more toxic than functionalized ones regardless of the surface function; while non-porous ones have been found to be more toxic than porous ones^8^. Hemocompatibility tests have revealed these observations being due to silanol groups being able to interact specifically with choline headgroups of the phospholipids on the cell surfaces^9^, an interaction which is prevented when the silica surface is functionalized. Due to the same reason, MSNs have shown lower hemolytic activity than their non-porous counterparts of similar size, as fewer silanol groups are available on the cell-contactable porous surface^10^. Erythrocytes that are not capable of active uptake are thus a good cell model to study interactions at the nano-bio interface, but it should be noted that observed toxicities are not only cell-line dependent but also assay dependent^11, 12^.

Surface charge not only determines the interaction of NPs with cell membranes, but also affect the biodistribution and residence time in the organism. Balogh *et al*.^13^ encapsulated AuNPs of different sizes into dendrimers, providing negative and positive charges to the different composites. Results showed that the particles selectively accumulate in different organs depending either on size or charge alone. For instance, when Balogh *et al*. compared particles of the same size, and showed that positively charged particles persist in the kidneys for up to four days (and finally excreted mostly by urine), whereas the negatively charge particles, and neutral particles remained in the liver and spleen^13^.

Thus, toxicity needs to be evaluated in model systems, which are relevant and predictive of human physiology, yet convenient, affordable, fast and ethical to allow for thorough and systematic evaluation at a high scale. The zebrafish (*Danio rerio*) is rapidly becoming an established animal model system to study nanoparticle toxicity^14, 15^. The zebrafish is small in size, have an efficient reproducibility and a fast embryonic development^16^. Further, it is transparent and the development of organs and tissues can be readily visualized. Different parameters can be assessed, such as hatching, organ development, (swimming) behavior, immunotoxicity, and genotoxicity, in addition to reproductive toxicity and mortality^16^. Another advantage of the zebrafish is that many organ systems such as the cardiovascular^17^, nervous and digestive systems of this model are similar to mammals^14^. The zebrafish is therefore a relevant model to analyze toxic disruption in endocrine, cardiovascular and neuronal function^18^. Further, the transparency of the zebrafish allow for detailed analyses of particle biodistribution *in vivo* ^19^.

To date, the zebrafish has mainly been used as an *in vivo* model to evaluate the toxicity of selected therapeutic MSNs, with a specific emphasis on the effect of the particles on adult or embryonic mortality^20, 21^. A few studies have used the model to assess *in vivo* delivery potential^22^, or therapeutic efficacy. In this respect, the zebrafish was recently used to evaluate mesoporous silica-coated upconversion nanocrystals for light triggered control of gene expression (knock down and inducible expression by photomorpholinos)^23^. Another study assessed MSNs in sensing of hydrogen peroxide and treatment during heart failure^24^. However, despite the obvious power of the model, the zebrafish has to date not been used to evaluate tissue penetrance and toxicity of differently functionalized MSNs in a systematic manner to understand chemical design-toxicity relationships. One study addressed the relevance of surface charge on the development of the so called protein corona^25–28^, a protein coat of the particles, which is formed upon exposure to protein rich biological fluids and critically influences biodistribution, therapeutic efficacy and toxicity. This was done by pre-incubating the particles with serum and then exposing the zebrafish to particles that had been or had not been incubated in serum^29^. They demonstrated that strongly positively charged MSNs caused 94% of the zebrafish embryos to die. However, there is no report on a systematic comparison of the surface functionalization-dependent biocompatibility and distribution of MSNs using the zebrafish as a model.

## Materials and Methods

### Synthesis of mesoporous silica nanoparticles

**Figure 1:**
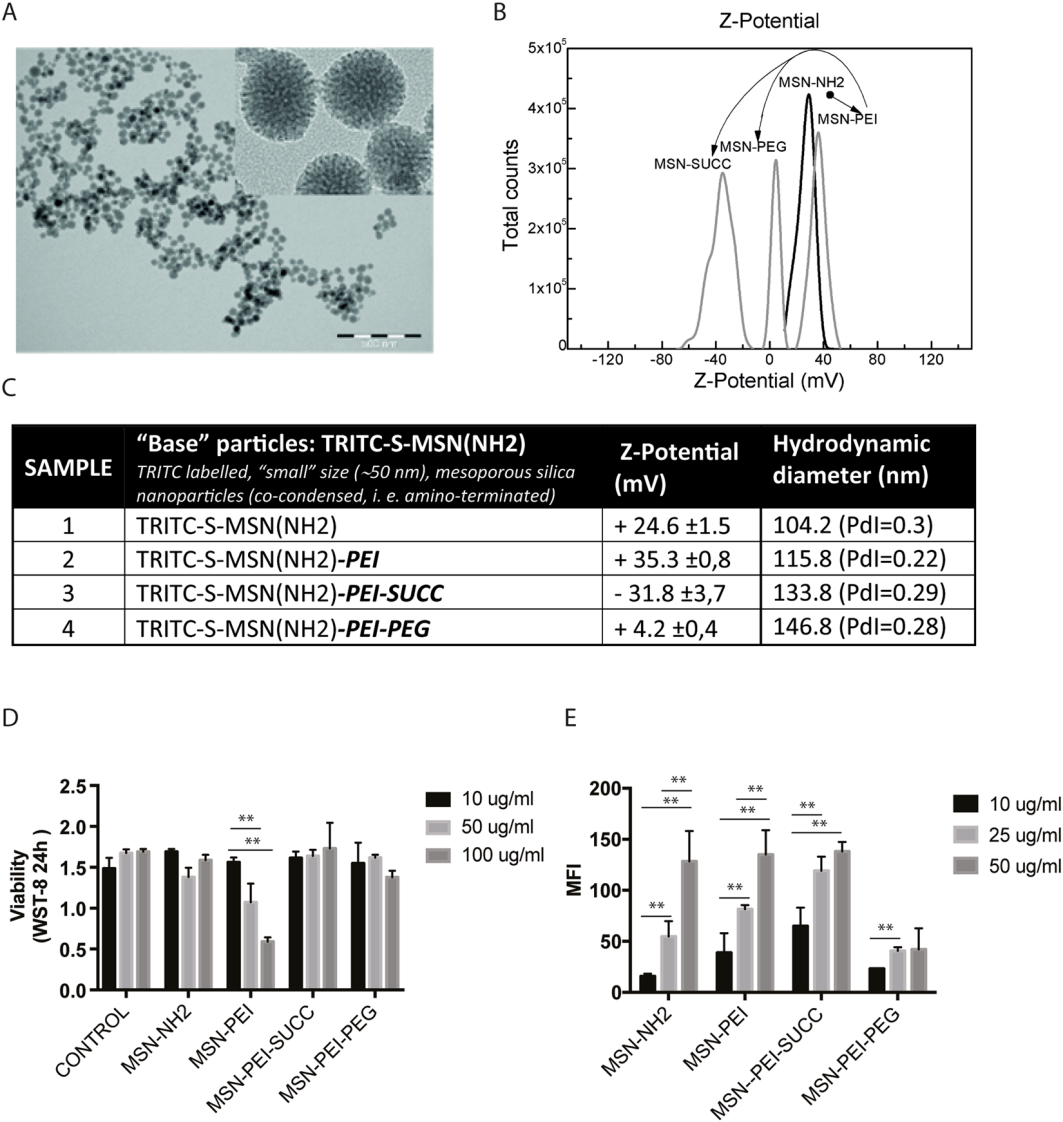
Characterization of the MSNs used in this work. (**A**) Representative TEM images and size distribution of the base MSN-NH_2_ particles. (**B**) Successful functionalization is verified by ζ-potential measurements demonstrating narrow charge distribution for each functionalization step. The arrows indicate the sequence of functionalization steps. (**C**) Table of the characteristics of the MSNs used in this work. Measurements have been done in HEPES buffer (25 mM) at pH = 7.2. Standard deviation in ζ-potential measurements is from three different measurements on the same sample. DLS measurements have been performed also three times and averaged polydispersity index (PdI) is also provided. Slight increase in DLS values and maintenance of the PdI indicates also that MSN maintain their size distribution after the successful functionalization steps. (**D**) Fluorescently labeled MSNPs were suspended in growth medium at a concentration of 10, 25 and 50 μg/ml The amount of endocytosed particles inside cells was analyzed by r flow cytometer. The mean fluorescence intensity of the cells at FL-1 channel is shown (n ≥ 3 ± SEM, *p < 0.05, **p < 0.01 ***p < 0.001). (**E**) The *in vitro* cytotoxicity of the MSNs in C2C12 myoblast cells was assessed using a WST-8 reagent. Absorbance at 450 nm is shown (n ≥ 3 ± SEM, *p < 0.05, **p < 0.01 ***p < 0.001).

The differently charged mesoporous silica nanoparticles (MSNs) used in this work were synthesized by wet chemistry methods under conditions known to provide stable and narrowly dispersed nanoparticles. Figure 1 shows the characteristics of the employed MSNs. Unless otherwise noted, all reagent-grade chemicals were purchased from Sigma-Aldrich and used as received. Millipore water was used in the preparation of all aqueous solutions. To facilitate the reading, acronyms of the reagent are as follows: Cetylmethylammonium bromide (CTAB); Anhydrous dimethyl formamide (DMF), ethylene glycol (EG), tetraethyl orthosilicate (TEOS), 3-aminopropyltriethoxysilane (APTES), poly(ethylene glycol) methyl ether (mPEG; 5000 mW). Polyethylenimine (PEI), Ammonium hydroxide (NH4OH), tetramethylrhodamine isothiocyanate (TRITC).

The synthesis of the MSN-NH_2_ was carried out by using a similar protocol as in our previous studies^30^. Specifically, MSN-NH_2_ were prepared by the co-condensation procedure where APTES and TEOS served as the precursor of silica. Fluorescent labelling of particles is carried out by using TRITC, an amine reactive fluorophore, by a pre-reaction of the TRITC with 0.3 mL APTES, to create inherently fluorescent particles. This reaction is done in 2 ml of ethanol and at 1:3 TRITC:APTES molar ratio, and stirred during 3 hours under inert atmosphere. To synthesize the MSN-NH_2_, a solution of CTAB (0.45 g, serve as template for the silica condensation) in a solution of 150 mL of water and 30 mL of ethylene glycol is heated to 70 °C in a three-neck round bottom flask of 250 mL capacity. When the temperature is reached ammonium hydroxide (30 wt%, 2.5 mL, serve as catalyst for the silica condensation), TEOS (1.5 mL) and the TRITC pre-reacted APTES (0.3 mL) are sequentially added to the solution under stirring. Thus, the molar ratio used in the synthesis is calculated as 1TEOS: 0.19APTES: 0.18CTAB: 5.9NH_4_OH: 88.5EG: 1249H_2_O. The mixture continues under stirring for 3 hours at the 70 °C. Then, stirring is stopped and the colloidal is aged at 70 °C overnight. Afterwards, temperature is stopped and the solution is allowed to cool down. Finally, efficient template removal is essential in order to avoid the interference of CTAB, a known toxic detergent, in the biological experiments. To this, a highly efficient ion-exchange method is carried out^31^, where the as-synthesized MSN-HH_2_ are purified by means of centrifugation (10.000 *g*, 10 min) and resuspended in a “template removal” solution, containing 60 mg ammonium nitrate in 20 mL of ethanol. This centrifugation and resuspension process is performed three times and the final products are resuspended in ethanol further use.

The preparation of the MSN-PEI was done by hyperbranching surface polymerization from aziridine monomer as also described in our previous works^32, 33^. Briefly, the MSN-NH_2_ “base” particles were transferred to toluene, followed by the addition of aziridine in the presence of catalytic amounts of acetic acid at room temperature and under argon atmosphere. The solution was left under stirring overnight, and further washed two times in ethanol (centrifuged at 10000 *g* 10 minutes) and finally the resulting MSN-EPI were resuspended in ethanol at the desired concentration for the biological experiments.

The preparation of MSN-SUCC was done starting with 10 mg of the MSN-PEI particles in 10 mL of ethanol, and adding 100 mg of succinic anhydride to the suspension (100 wt% excess). The mixture was stirred at room temperature for 6 h and finally purified and resuspended in ethanol as explained above.

Finally, MSN-PEG samples were obtained as described in ref. 34. This is two-step process. First, activation of the mPEG with catalytic amounts of hexamethylene diisocyanate (HMDI) in chloroform. Once the mPEG is activated and dried, it is added also in 100 wt% excess to a solution of MSN-PEI in chloroform and allowed to conjugate overnight. Finally, the suspension was washed two times and resuspended in ethanol.

### MSN characterization

MSN dispersions were fully characterized by means of different techniques such as Transmission Electron Microscopy (TEM), Zeta Potential (ζ-Potential) and Dynamic Light Scattering (DLS). For ζ-potential and DLS measurements, the samples were dispersed at a mass concentration of 1 mg/ml in HEPES buffer (25 mM; pH = 7,4) and measured immediately. The suspersions were stable throughout the characterization process.

### Transmission Electron Microscope (TEM)

TEM images were acquired with a TEM images were acquired with a JEM 1400-Plus (JEOL Ltd., Tokyo, Japan) operating at 80 kV with a tungsten filament and an 11-Mpx CCD camera. MSN samples were prepared by drop casting onto the carbon coated cooper TEM grids. Observations were made with different magnifications at different parts of the grid for obtaining of statistically significant results of MSN size distribution, analyzed using ImageJ software.

*Zeta-Potential measurement (ζ-Potential) and Dynamic Light Scattering (DLS)* Measurements were made with a Malvern ZetaSizer Nano ZS Instrument operating at a light source wavelength of 532 nm and a fixed scattering angle of 173° for detection. 0.8 ml of the colloidal suspensions of NPs were placed into the specific cuvette and the software was arranged with the specific parameters of refractive index and absorption coefficient of NPs material and solvent viscosity, data required to obtain the correct value for each type of NP.

### Cellular model systems and cell culture conditions

Mouse derived C2C12 myoblasts (including stable and inducible cell lines) were grown in DMEM (Sigma D6171) supplemented with 10% FCS (PromoCell C- 37360), 1 mmol L-glutamine (Sigma G7513), 25,000 units penicillin/25 mg streptomycin (Sigma P0781), nonessential amino acids (Sigma M7145), and sodium pyruvate (Sigma S8636).

### Flow cytometry to analyse the uptake of nanoparticles ***in vitro***

Fluorescently labeled MSNPs were suspended in growth medium at a concentration of 10, 25 and 50 μg/ml. After 20-minute sonication in water bath, the medium with particles or the control medium was added to the 50–70% confluent cells and incubated for 24 hours at 37 °C. The cells were trypsinized and the extracellular fluorescence was quenched by resuspension to 200 μg/ml trypan blue (Fluka, St Louis, MO) for 5 minutes at room temperature. The cells were washed once and resuspended in phosphate-buffered saline. The amount of endocytosed particles inside cells was analyzed by FacsCalibur flow cytometer (BD Pharmingen, San Diego, CA). The mean fluorescence intensity of the cells at FL-1 channel was measured. The data were analyzed with Cyflogic software.

### Metabolic assay to analyse the influence of nanoparticles on viability *in vitro*

The cytotoxicity of the nanoparticles was assessed using a WST-8 kit, according to the manufacturer’s manual (#CK04–11, Dojido Molecular Technologies, Inc). In brief, C2C12 myoblast cells were seeded in a 96-well plate at a density of 5000 cells/well. After allowing time to attach, the medium was exchanged for medium containing different concentrations of nanoparticles (10, 50 and 100 µg/ml). The cells were incubated for 22 hours, whereafter 10 µl of CCK-8 solution was added to each well, and cells were allowed to incubate for an additional 2 hours. The absorbance was measured at 450 nm using a microplate reader.

### Zebrafish embryo toxicity assay

All zebrafish experiments were performed according to relevant Swiss and Finnish legislature, and adult fish were maintained under licenses 1014H and 1014G1 issued by the Veterinäramt-Basel-Stadt, Switzerland and *MMM/465/71*2*-93 issued by Ministery of Agriculture and Forestry, Finland*. Zebrafish embryos were obtained by natural spawning. Embryos were collected from breeding tanks and washed with E3-medium (5 mM NaCl, 0.17 mM KCl, 0.33 mM CaCl_2_, 0.33 mM MgSO_4_). Embryos were cultured at 28.5 °C in E3 medium. Treatment with nanoparticles were started at 6hpf or 24hpf. Nanoparticle solutions were vortexed vigorously or sonicated (10 min, Branson sonicator, model 2210) prior to dilution in E3 and administration to the embryos. In all treatments the final concentration of DMSO was 1%. In some experiments, the chorion membrane was removed by enzymatic treatment with pronase (1 mg/ml) followed by extensive washes with E3 before exposure to nanoparticles. Embryos were cultured in 96-well plates, one embryo per well. The embryos were analysed using stereomicroscope and the condition of embryos (live/dead or blood flow) was assessed visually. The cardiovascular function was visually assessed in qualitative manner using a stereomicroscope.

### *Microinjection of zebrafish embryos*

Glass capillary needles were manufactured using glass capillaries (GC-100TF, Company) and vertical needle puller (model PB-7, Narishige). Solutions were injected into 4hpf zebrafish embryos using Femtojet microinjector (Eppendorf) and Transferman (Eppendorf) micromanipulator mounted to SteroLumar V12 stereomicroscope (Zeiss). The equipment was calibrated using injections of TMR_dextran (0.5 mg/ml in 90% DMSO, 2000 kDa TMR-dextran, Thermo Fischer) into halocarbon oil (Halocarbon oil 27, H8773, Sigma-Aldrich) and in into embryos followed by measurement of fluorescence intensities. Calibration by injections into halocarbon oil indicated delivery of 1.0 +/− 0.06 nl (n = 10), but fluorescence measurement from the embryos indicated that actual amount delivered *in vivo* was 2.0 +/− 1.2 nl (n = 10). In the toxicity experiment, either DMSO alone or MSNs dissolved in DMSO (10 mg/ml) were injected. After injections, damaged embryos were removed and healthy embryos were cultured in E3 at 28.5C.

### *Live confocal imaging of zebrafish embryos*

For imaging, pigment formation of zebrafish embryos expressing EGFP in the vasculature (Tg kdrl:EGFP s843)^35^. was inhibited by culturing them in the presence of PTU (concentration 0.2 mM, 4-phenyl-2-thiourea, Sigma). At age 72hpf the embryos were anesthesized using Tricaine (160 mg/ml, Sigma) and subsequently, the embryos were mounted into low-melting point agarose on a glass-bottom dishes (Mat-Tek). Agarose was overlaid with E3 supplemented with tricaine and PTU. Live embryos were imaged using Leica Sp5 Matrix confocal microscope (10x objective). Image processing was performed by using Fiji software^36^.

### Statistical analyses

**Figure 2:**
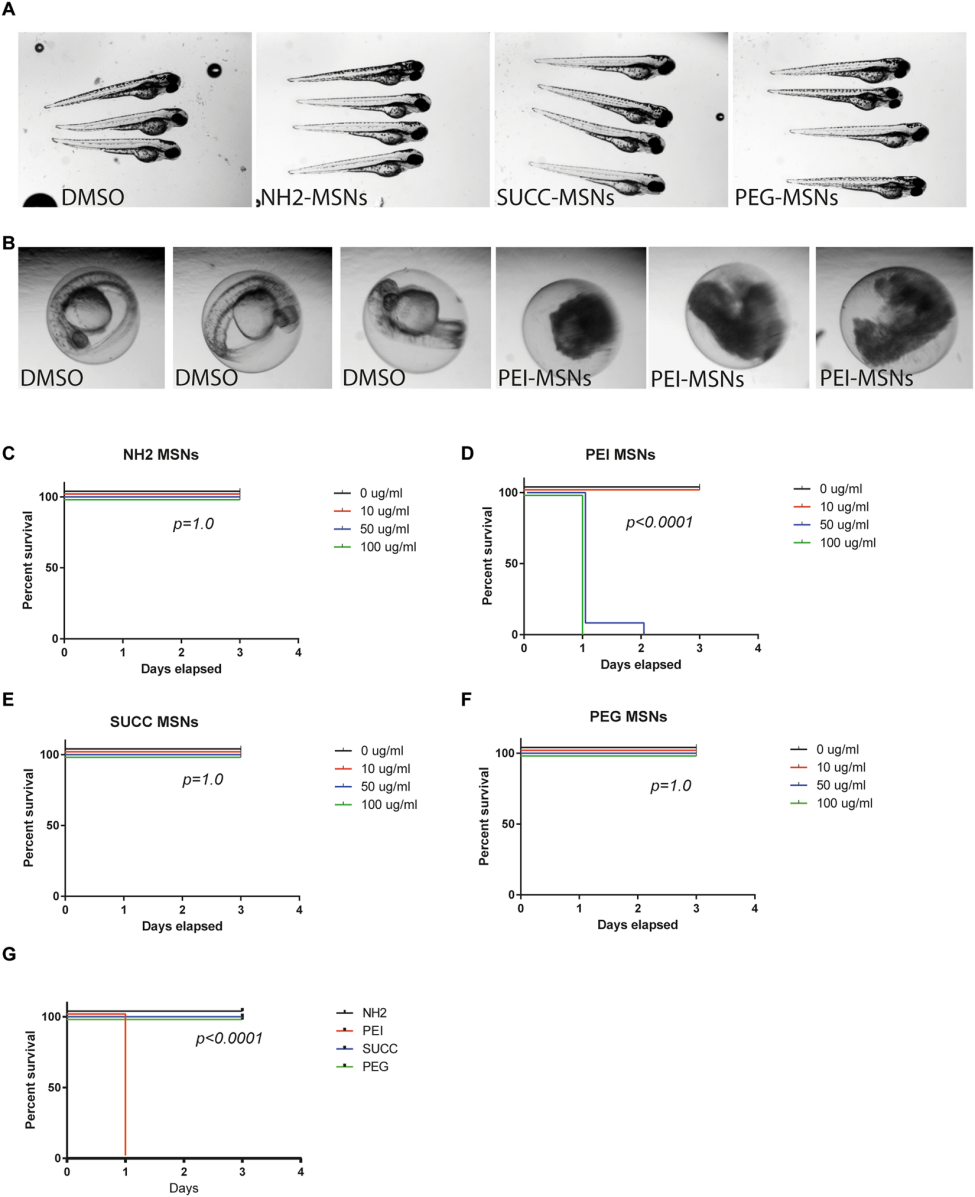
Surface functionalization affects overall toxicity of MSNs. (**A**) Zebrafish embryos at 72 hpf treated with the DMSO vehicle, NH2-, SUCC- and PEG-MSNs at a concentration of  50 μ/ml. Treatment was initiated at 8hpf. The embryos are anesthesized and mounted in 2%methyl cellulose. (**B**) Zebrafish embryos at 48 hpf treated the DMSO vehicle or  50 μg/ml PEI-MSNs. Treatment was initiated at 8hpf. (**C**–**F**) Percentage of survival of embryos at 24hpf, 48 hpf and 72hpf upon treatment with (**C**) NH_2_-, (**D**) PEI-, (**E**) SUCC- and (**F**) PEG-MSNs at denoted concentrations. The treatment was initiated at 8hpf. N = 24 embryos/treatment, except in PEI-MSN 10 μg/ml (n = 25) and SUCC-MSN 100 μg/ml (n = 25). Each embryo was followed throughout the experiment and score live or dead on each day (**G**) Comparison of survival between nanoparticles at 100 μg/ml concentration.

**Figure 3:**
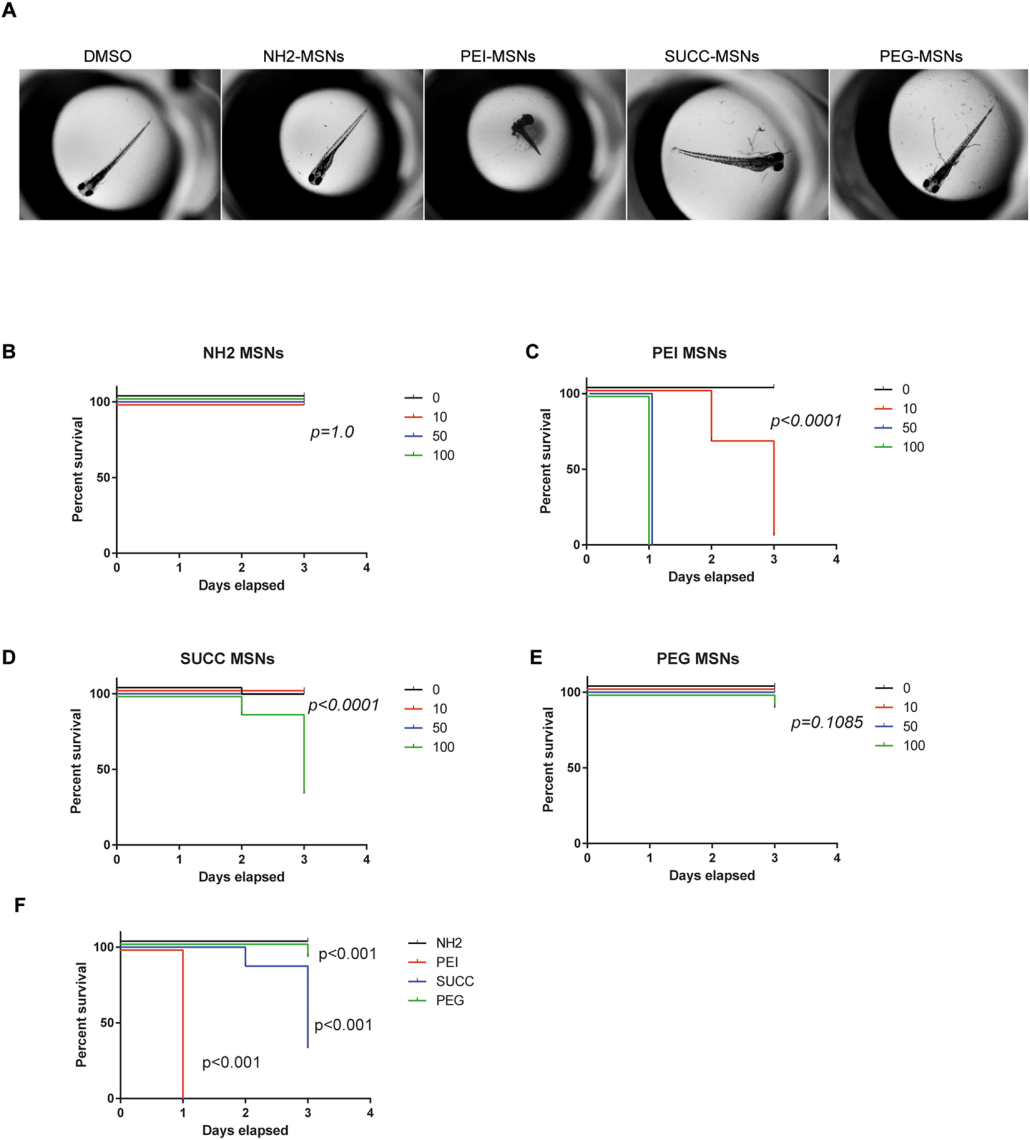
Dechorionation sensitizes embryos for MSN induced toxicity. (**A**) Images of dechorionated zebrafish embryos at 96 hpf after treatment with NH2. PEI, SUCC and PEG.-MSNs. The treatment was initated at 24hpf. (**B**–**E**) Percentage of survival of dechorionated embryos at 48 hpf, 72hpf and 96 hpf upon treatment with (**B**) NH2, (**C**) PEI, (**D**) SUCC and (**E**) PEG MSNs at denoted concentrations. The treatment was initated at 24hpf. N = 24 embryos/treatment, except in PEI-MSN 0 μg/ml (n = 25), SUCC-MSN 0 μg/ml (n = 23), SUCC-MSN 10 μg/ml (n = 23) and SUCC-MSN 100 μg/ml (n = 25). Each embryo was followed throughout the experiment and score live or dead on each day (**F**) Comparison of survival between MSNs at 100 μg/ml concentration.

**Figure 4:**
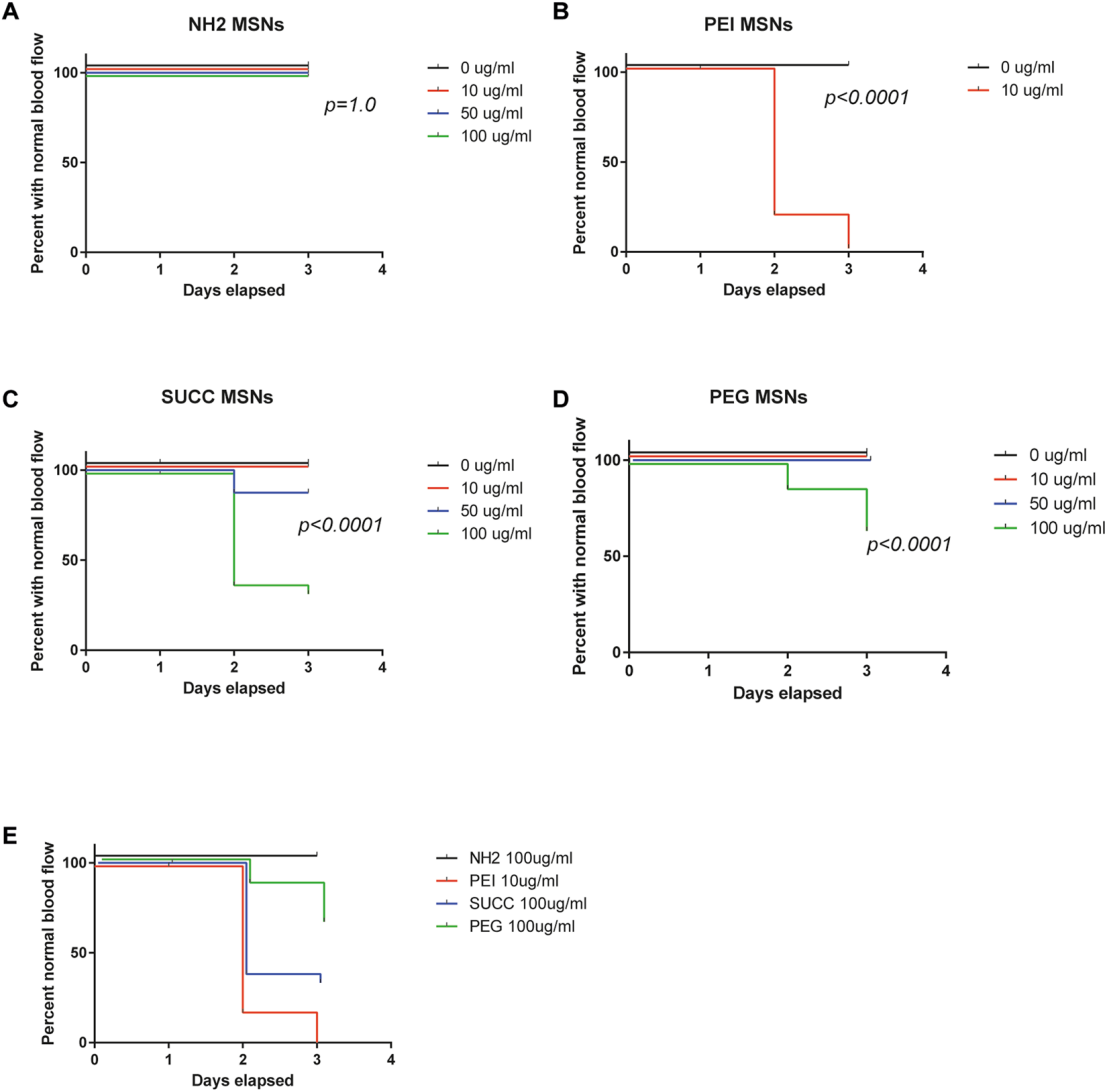
Surface functionalization affects cardiovascular toxicity of MSNs. The graphs show percentage of dechorionated embryos that show normal blood circulation upon treatment with NH2. PEI, SUCC and PEG MSNs at denoted concentrations. The treatment was initated at 24hpf and cardiovascular function analysed at 72 and 96 hpf. N = 24 embryos/treatment, except in PEI-MSN 0 μg/ml (n = 25), SUCC-MSN 0 μg/ml (n = 23), SUCC-MSN 10 μg/ml (n = 23) and SUCC-MSN 100 μg/ml (n = 25). Higher concentrations (50 and 100 μg/ml) of PEI-MSNs were not assessed due to high lethality. Each embryo was followed throughout the experiment and cardiovascular function was visually scored normal or abnormal day 3 and 4. G) Comparison of survival between MSNs at highest used concentration.

**Figure 5:**
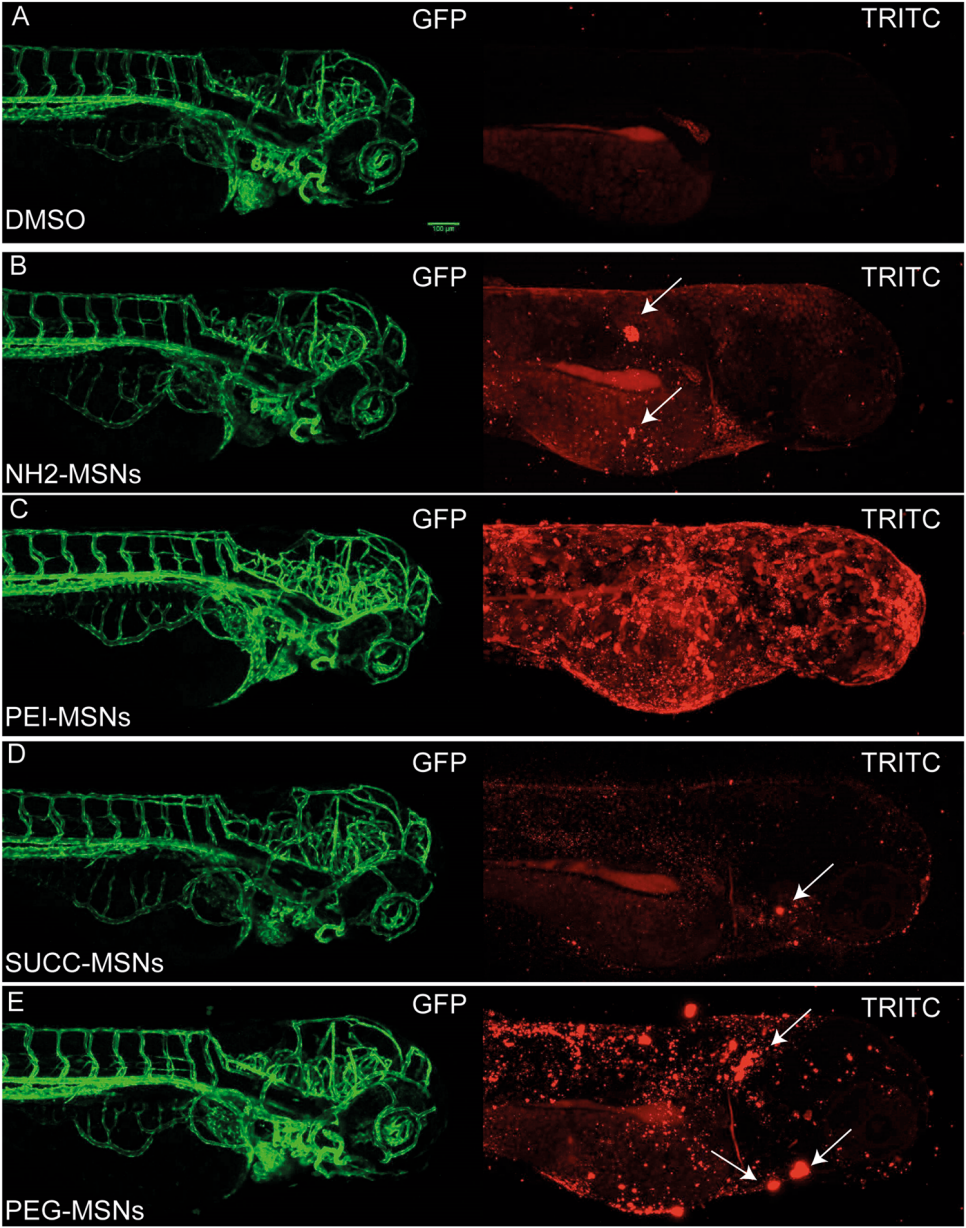
Surface functionalization affects aggregation of MSNs on the embryo skin. The uptake of the nanoparticles from the aquaeous medium into the embryo was analyzed by mounting anesthesized living embryos into agarose and imaged using confocal microscopy (Leica SP5 Matrix). A transgenic strain (kdrl:EGFP) expressing a GFP labeled vasculature were used 24hpf embryos were incubated 48 h with the highest tolerated dose of the different nanoparticles e.g 100 μg/ml NH2-, SUCC- and PEG-MSNs, and 10 ug/ml of PEI-MSNs. DMSO treated embryos were used as controls and show background fluoresence (**A**). Maximum Z-projections are shown. The white arrows mark nanoparticle clusters on the superficial layer of the embryo.

**Figure 6:**
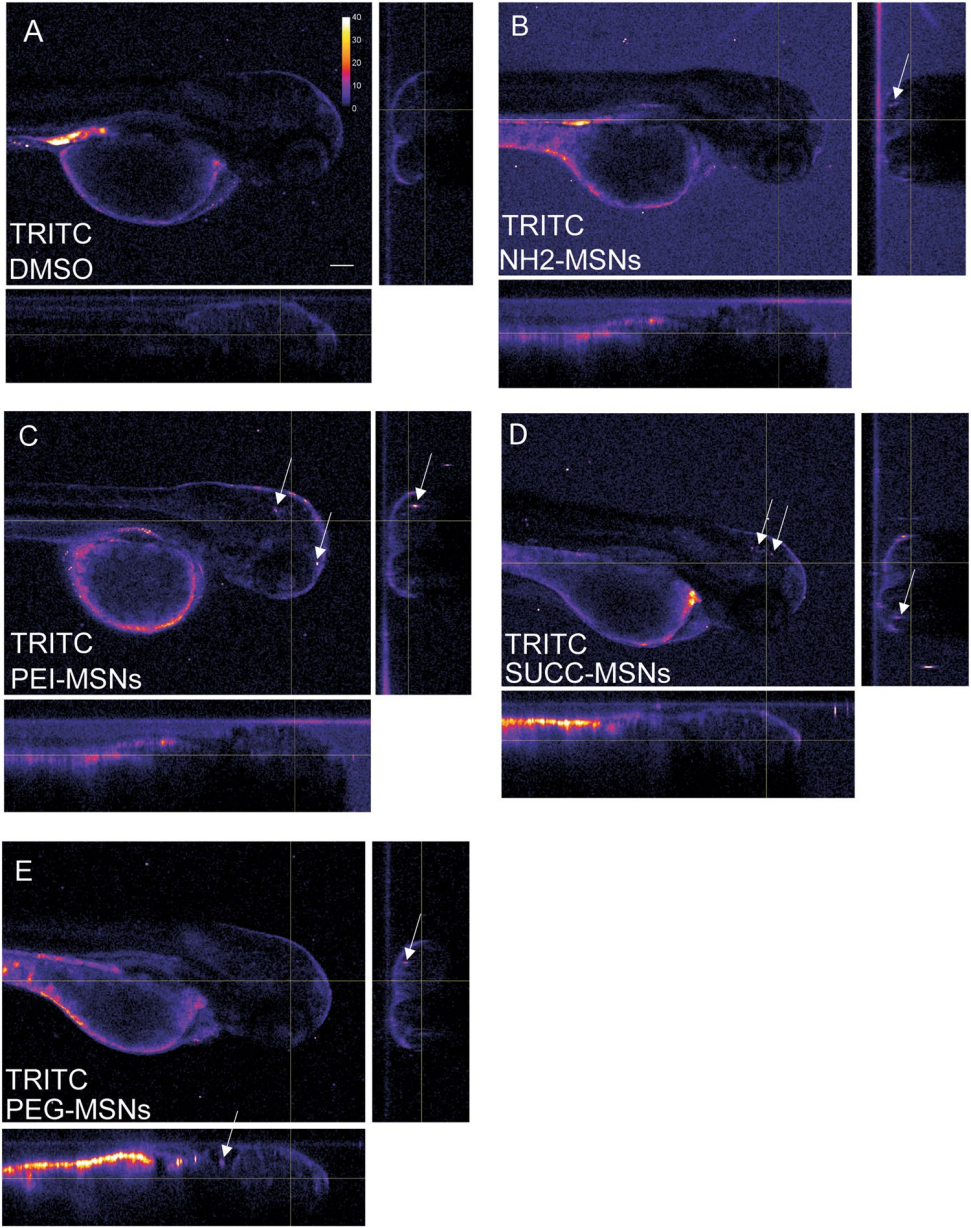
Surface functionalization affects uptake of MSNs. The uptake of the nanoparticles from the aquaeous medium into the embryo was analyzed by mounting anesthesized living embryos into agarose and imaged using confocal microscopy (Leica SP5 Matrix). A transgenic strain (kdrl:EGFP) expressing a GFP labeled vasculature were used. 24hpf embryos were incubated for 48 h with the highest tolerated dose of the different nanoparticles e.g 100 μg/ml NH2-, SUCC- and PEG-MSNs, and 10 μg/ml of PEI-MSNs. DMSO treated embryos were used as controls and show background fluorescence (**A**). Ortohogonal views of the 3D confocal data sets are shown to visualize fluorescence deep inside the embryo. Panels are pseudocolored for clearer visualization of nanoparticle fluorescence in the brain. Calibration bar in panel 6A indicates relation between colour and intensity values. White arrows mark nanoparticle clusters inside the embryo brains. Scale bar is 100 µm.

**Figure 7:**
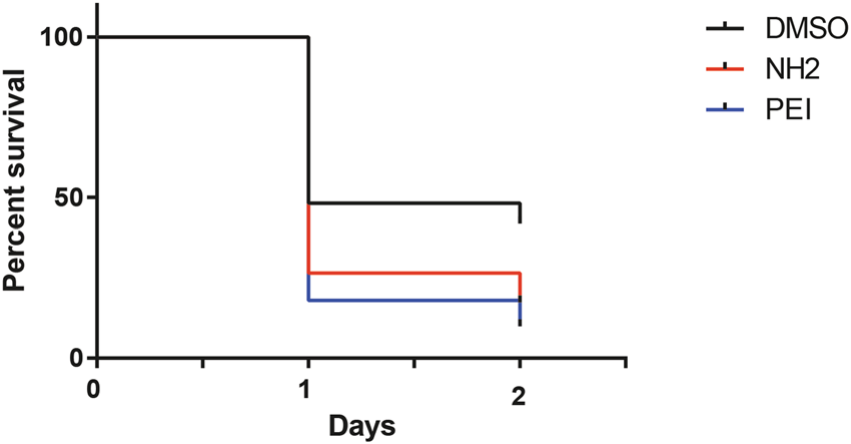
Bypass of biological barriers by microinjection increases toxicity of NH2-MSNs. Percentage survival of zebrafish embryos microinjected with 20+/−12 ng/embryo NH_2_-MSNs (n = 111) and PEI-MSNs (n = 98), or with DMSO control alone (n = 110).

Statistical survival analyses were performed using Log-rank (Mantel-Cox) test and computed with GraphPad Prism version 6.05 for Windows (GraphPad Software, La Jolla, California, USA). P-values were adjusted due to multiple tests using Bonferroni correction. Cases where any of the curves show no toxicity, p-value cannot be computed. As curves are identical, they were given maximal p-value (p = 1.0). In each exposure experiment (Figs 2–4), 23–25 embryos were individually treated and maintained as single embryos in 96-well plates. In the imaging experiments (Figs 5 and 6), 5 embryos/treatment was imaged using confocal microscope. In the microinjection experiments (Fig. 7), data from two separate experiments with embryos from different clutches were combined (total n = 98–111/treatment).

## Results

### *Functionalization of MSNs to obtain particles with different surface charge*

The MSNs were derivatized from the same core particle, TRITC-labelled ~50 nm MSNs co-condensed with an aminosilane (MSN-NH_2_), which were further functionalized to provide the different surface charges. In essence, the “base” particles (MSN-NH_2_) were functionalized with polyethyleneimine (MSN-PEI), to provide the maximal amount of amine groups and a positive charge, succinic anhydride (MSN-SUCC) to provide negatively charged succinic acid groups and polyethyleneglycol (methyl ether) mPEG, 5 kDa, providing neutral charge (MSN-PEG). Transmission Electron Microscopy (TEM) was used to analyze the size distribution of the “base” MSN-NH_2_ and to confirm that no further aggregation occurred after functionalization (Fig. 1A). The success of the functionalization was also assessed by the increase in the hydrodynamic diameter (Fig. 1C), measured by Dynamic Light Scattering (DLS), and the modification of the MSN surface charge, analyzed by ζ-potential measurements (Fig. 1B and C). The narrow charge distributions after the different modifications and the slight increase in DLS values along with the maintenance of the polydispersity index indicates that MSN maintain their size distribution after the successful functionalization steps (Fig. 1C).

### *Polyethylenimine (PEI) and amino (NH*_2_*) functionalized MSNs demonstrate enhanced cellular uptake in vitro, as compared to Polyethylene glycol (PEG) and Succinyl (SUCC) functionalized particles*

C2C12 myoblasts were grown to 70% confluency, whereafter they were incubated with 10, 50 or 100 µg/ml PEI (PEI-MSN), NH_2_ (NH_2_-MSN), PEG (PEG-MSN) and SUCC (SUCC-MSN) functionalized TRITC-labeled MSNs in the presence of serum for 24 h. Cellular uptake was assessed by flow cytometry. All particles were internalized in a dose dependent manner with increasing concentration correlating with increasing uptake (Fig. 1D). The PEI- and NH_2_-MSNs showed a significantly higher uptake than PEG-MSNs and SUCC-MSNs (Fig. 1D).

### PEI functionalization decreases viability in myoblasts in a particle concentration dependent manner

We next evaluated toxicity of the particles by the WST-8 viability assay, using the same concentration range, 10, 50 or 100 µg/ml. C2C12 cells tolerated the particles well. PEI-MSNs induced a concentration dependent reduction in viability after 24 h. A 20–30% reduction was observed at 50 µg/ml whereas a 50% reduction in viability was observed at 100 µg/ml (Fig. 1E).

### *High concentrations of PEI-MSNs but not PEG, NH*_*2*_*and SUCC-MSNs leads to enhanced mortality in zebrafish embryos*

We next utilized the zebrafish as a model to evaluate toxicity of the functionalized MSNs *in vivo*. Zebrafish embryos were exposed of 0, 10, 50 or 100 µg/ml of the PEI, PEG, NH_2_ and SUCC functionalized nanoparticles. We used 24 embryos per concentration and treated the embryos starting from 8 hours post fertilization (hpf). The embryos were analyzed at 24, 48 and 72 hpf. No obvious effects were observed on embryos treated with PEG, NH_2_ and SUCC and the embryos developed normally until the experiment was terminated (Fig. 2A,C,E,F). On the contrary, the PEI-MSNs demonstrated significant toxicity and the embryos treated with 50 µg/ml PEI particles were dead by 48 hpf (Fig. 2B and D). Direct statistical comparison of different MSNs at highest concentrations indicated that PEI-MSNs showed clear toxicity when compared to NH2-MSNs (p < 0.0001, Fig. 2G).

### Removal of the chorion membrane sensitizes zebrafish embryos to MSN toxicity

Next we removed the protective chorion membrane around the embryo to allow better penetration of all the differently surface-functionalized nanoparticles. We initiated the treatment of the dechorionated embryos at 24 hpf and analysed the embryos at 48, 72 and 96 hpf. This experiment verified the toxicity of the PEI functionalized particles (Fig. 3A–F). The dechorionated embryos were more sensitive to PEI-MSNs and a reduction in viability was evident already at a concentration of 10 µg/ml upon prolonged treatment (Fig. 3C and F). The viability of embryos treated with 10 µg/ml was 100% at 48hpf but decreased to 80% at 72hpf and was less than 5% at 96hpf (Fig. 3C). The embryos tolerated the PEG-, NH_2_- and SUCC-MSNs well and only upon prolonged incubation (96 hpf) at high concentration (100 µg/ml) a reduction in viability to 50% was observed for the PEG- and SUCC-MSNs (Fig. 3E). Direct statistical comparison of different MSNs at highest concentrations indicated that PEI-MSNs was the most toxic (p < 0.0001) and also SUCC-MSNs exhibited clear toxicity (p < 0.0001) when compared to NH_2_-MSNs (Fig. 3F).

### PEI functionalized particles impair cardiovascular function

We also assessed cardiovascular function using a stereomicroscope as blood circulation can be easily observed in the embryo (Video 1). Blood circulation was analysed, by bright field illumination, at 72hpf and 96hpf old embryos treated with 0, 10, 50 or 100 µg/ml of PEI, PEG, NH_2_ and SUCC functionalized particles. The treatment was initiated at 24hpf. In line with the data on embryo viability, the PEI-MSNs had the most disruptive effect on the embryos and only 20% of the embryos treated with 10 µg/ml of PEI-MSNs showed a normal circulation at 72hpf (Fig. 4). At higher concentrations and increased duration of treatment, all embryos were affected (Fig. 4). Also SUCC- and PEG-MSNs treated embryos demonstrated a disrupted circulation at high concentrations upon prolonged exposure, where the effect of SUCC-MSNs was more pronounced. PEI-MSNs showed significant cardiovascular toxicity already at lowest tested concentration (Fig. 4B). Less than 30% of embryos treated with 100 µg/ml SUCC-MSNs showed normal circulation at 72hpf and 96hpf (Fig. 4C). 100 µg/ml PEG-MSNs affected approximately 20% and 30% of the embryos at 72hpf and 96hpf respectively (Fig. 4D). NH_2_-MSNs did not show any adverse effects within the time frame and at the concentrations tested (Fig. 4A). Direct statistical comparison of MSNs at highest analysed concentration indicated significant cardiovascular toxicity of 10 ug/ml PEI-MSNs and also 100 ug/ml SUCC-MSNs (Fig. 4E).

### PEI-MSNs shows the most efficient penetration into the zebrafish embryo

We next analysed the uptake of the nanoparticles from the aqueous medium into the embryo by mounting anesthetized living embryos, that had been incubated with nanoparticles, into agarose and imaging them using confocal microscopy. We exposed transgenic zebrafish embryos (kdrl:EGFP) with a GFP-labeled vasculature to the highest tolerated dose of nanoparticles for 48 h. The used concentrations were 100 µg/ml for the NH_2_-, SUCC- and PEG-MSNs, and 10 µg/ml for the PEI-MSNs. All particles aggregated onto the surface of the embryos, as visualized by maximum Z-projections of 3D confocal imaging data (Fig. 5A-E). The PEG-MSNs showed a clearly more pronounced aggregation (Fig. 5E), whereas SUCC-MSNs (Fig. 5D) show a low amount of accumulated particles amount of nanoparticle accumulation. NH2-MSNs and PEG-MSNs showed heterogenous clusters (Fig. 5B and E), whereas in PEI-MSNs the pattern is more homogenous (Fig. 5C).

To unambiguously identify nanoparticle clusters deep inside the embryo, we analysed the 3D confocal imaging data using orthogonal views (Fig. 6A–E). At 48 h of incubation the PEI particles had penetrated into the embryo (Fig. 6C). Also the other MSNs demonstrated a slight penetration into the embryo (Fig. 6B,D and E). Although the highest fluorescence was still observed in the skin and quite little fluorescence was observed in the deeper tissues (Fig. 6A–E).

### The toxicity of MSNs depends on the administration route and is enhanced when particles are microinjected into the embryo

The imaging data indicated that differences in the extent of particle penetration into the embryos might explain the differential toxicity. To further elucidate this, we evaluated the effect of the PEI- and NH_2_-MSNs, the particles that so far had demonstrated the highest and lowest toxicity, upon microinjection into zebrafish embryos. The embryos were microinjected with 20 ng of particles per embryo. MSNs were diluted in DMSO. At the used DMSO concentration the vehicle caused a 50% mortality rate and hence the concentration of particles could not be increased. Both particles demonstrated a clear reduction in viability at 24hpf and 48hpf as compared to the DMSO control (p = 0.0003 and p < 0.0001), whereas there was no statistically significant difference between NH_2_-MSNs and PEI-MSNs (p = 0.305) (Fig. 7). The unexpected toxicity of the NH_2_-MSN upon microinjection suggests that it is the penetrance and exposure of inner organs to the particles that is the cause of the adverse effects, rather than the chemical modification, and that surface functionalization affects toxicity by influencing biodistribution and penetrance of biological barriers.

## Conclusions and Discussion

In this work we evaluated the effect of PEI, PEG, NH_2_ and SUCC functionalized MSNs on the survival and cardiovascular function of zebrafish embryos. We either incubated embryos with the chorion membrane intact or dechoroniated embryos in medium with particles or microinjected particles into the embryos, and assessed viability and blood circulation at different time points and different particle concentrations. The PEI-MSNs were more toxic than other particles in cells and *in vivo* when embryos were incubated with particles (Figs 1–4). This is in line with previous studies stating that positive surface chare promotes cellular uptake and, consequently, may simultaneously cause a disproportionate decrease in cell viability due to significantly more pronounced uptake^12^. It should be noted that efficient cellular uptake is a prerequisite for successful intracellular drug delivery, whereby a more pronounced uptake means the administered dose can be minimized. For non-colloidally stable NP systems, the NPs may aggregate already in the cell media and thus form aggregates that are too large for cellular internalization, but rather form aggregates that are stuck to the outer cell membrane. Such cells can still be erroneously detected as “positive” in uptake experiments leading to false estimates of extent of cellular internalization.

Besides colloidal stability, hydrolytic stability (or any other mechanism of degradation in the physiological environment) is another issue that needs to be taken into account in validating the nanotoxicology profile of NPs. Nanomaterials may be unstable in physiological media and in the bloodstream due to the ionic strength and protein content of these environments. If destabilized or modified, their initially designed properties can be lost. The most significant alterations affecting the biological fate and effects of MSNs in biological environments can be summarized as: i) the formation of a protein corona as a result of the adsorption of proteins onto the inorganic surface, ii) the detachment of conjugated molecules and/or MSN dissolution into ionic species and, iii) the agglomeration and aggregation of the MSNs^2^. We have previously studied the evolution of MSNs with the same surface functionalizations as used in the present study in different biological media^37^, whereby our results indicated that both the morphological integrity and the effective surface charge of the particles were still retained to a certain degree after one week of incubation in the studied conditions. Thus, we believe the time-frame of the current experiments can also be related to the initial design of the MSNs i.e. to the different surface functionalizations.

The skin forms a natural biological barrier protecting underlining tissues from damage. Potential toxicity might be related to exposure of inner organs to particles. Biological barriers constitute one of the main hurdles that may hamper successful drug delivery but that can be overcome by rational design of nanopharmaceuticals^38^. We demonstrated that the particles aggregated onto the skin surface of the embryos however upon prolonged incubation PEI-MSNs clearly penetrated into the embryo. To circumvent toxicity arising from differential tissue penetrance we microinjected particles into the embryos, a fairly routine method in zebrafish, but with no translation into an actual drug administration regime. Interestingly, both the NH2- and PEI-MSNs, which demonstrated the lowest and highest toxicity upon incubation were equally toxic upon microinjection. This indicates that direct organ exposure is a more determining toxic factor than surface chemistry, and that surface functionalization mainly affect toxicity by modulating the ability of particles to penetrate biological barriers. In a drug delivery setting, this would be a desirable trait in minimizing drug exposure as the dose can be lowered.

Taken together our data shows that administration route, dose and duration of treatment are critical parameters for toxicity and systematic evaluation of all parameters is necessary to draw conclusions on biocompatibility. This data further emphasises the need of careful evaluation of particles on the physiological functionality of tissues and organs in addition to evaluation of gross mortality.

All in all, surface functionalization is a powerful way of tuning biodistribution and drug delivery potential of nanoparticles. Here we show that functionalization critically determines penetrance of biological barriers and consequent toxicity, and highlight that surface tailoring to fit the given application and target tissue is a crucial step in the development of safer and sustainable nanotherapies.

## Electronic supplementary material

Supplementary video legend

Supplement video
