# Supplementary material for: Analyses in zebrafish embryos reveal that nanotoxicity profiles are dependent on surface-functionalization controlled penetrance of biological membranes
Source: Sci Rep. 2017 Aug 21;7:8423. doi: 10.1038/s41598-017-09312-z (PMC5566213; doi:10.1038/s41598-017-09312-z)
Supplement: Supplementary file 1 — Supplementary video legend [file 41598_2017_9312_MOESM1_ESM.docx]

**Supplementary information**

**Analyses in zebrafish embryos reveal that nanotoxicity profiles are dependent on surface-functionalization controlled penetrance of biological membranes**

Ilkka Paatero^1,3#^, Eudald Casals^4^, Rasmus Niemi^2,3^, Ezgi Özliseli^4^, Jessica M. Rosenholm^4^ and Cecilia Sahlgren^2,3,5,6#^

***Supplement video 1***. Blood circulation in the trunk of the 3 day old untreated zebrafish. The normal blood circulation can be seen as fast moving blood cells in the zebrafish embryo tail. The movie was imaged with Zeiss StereoLumar V12 stereomicroscope with 80x total maginifcation at 30 frames per second (fps), and is displayed at 20fps.
